# Self-Injurious Behavior in People with Intellectual Disabilities and Co-Occurring Psychopathology using the Self-Harm Scale: A Pilot Study

**DOI:** 10.1007/s10882-018-9614-0

**Published:** 2018-07-27

**Authors:** Kim J. H. M. van den Bogaard, Henk L. I. Nijman, Tom Palmstierna, Petri J. C. M. Embregts

**Affiliations:** 1 Department Tranzo, Tilburg School of Social and Behavioral Sciences, Tilburg University, Prof. Cobbenhagenlaan 125, 5037DB Tilburg, The Netherlands; 2 Dichterbij Science and Innovation, Gennep, The Netherlands; 3 Behavioural Science Institute (BSI), Radboud University, Nijmegen, The Netherlands; 4 Aventurijn – Fivoor, Forensic Psychiatric Institute, Den Dolder, The Netherlands; 5 Centre for Psychiatry Research, Department of Clinical Neuroscience, Karolinska Institutet, & Stockholm Health Care Services, Stockholm County Council, Stockholm, Sweden; 6 St. Olav’s University Hospital, Center for Research and Education in Forensic Psychiatry, Norwegian University of Science and Technology (NTNU), Trondheim, Norway

**Keywords:** Intellectual disability, Self-injurious behavior, Self-Harm Scale, Psychopathology, Structured clinical assessment

## Abstract

Self-injurious behavior (SIB) is one of the most detrimental behaviors for the person showing it, as well as for their environment. Nevertheless, structured clinical assessments of SIB are scarce. Staff completed a Self-Harm Scale (SHS) every time they witnessed SIB in clients with an intellectual disability (ID) and co-occurring psychopathology (*N* = 33). Descriptive statistics were conducted to explore the nature of the incidents of SIB and the characteristics of the people involved in the incidents. In 41 weeks, 104 SIB incidents were reported for 8 out of 33 clients (24%). Incidents were most prevalent on Mondays (23%). As far as the methods of SIB concerned, cutting was the most used method (63%). Clients who showed SIB differed significantly from clients who did not on gender, having a personality disorder and communicative abilities. This study was one of the few that used an incident-based record form to report SIB by direct observation. It is hoped that the SHS helps to gain more information about SIB, to improve individualized interventions. Further research is necessary to determine the psychometric properties and clinical utility of the scale.

## Introduction

Self-injurious behavior (SIB) can be defined as *behavior in which a person harms (or attempts to harm) oneself deliberately and physically,* with typical examples like head-banging, self-biting and self-scratching (Lowe et al. 2007), with prevalence rates of 4.89% in a population-based cohort of adults with ID (Jones et al. 2008). SIB puts the individual at risk in a physical, psychological, and social way. It intervenes significantly with the quality of life of an individual and can lead to: 1) physical impairments or even death (Emerson 1992; Klonsky 2007; Nissen and Haveman 1997), 2) increased psychotropic medication (Matson and Neal 2009; Robertson et al. 2005), 3) mechanical restraint and protective devices (Robertson et al. 2005), 4) feelings of shame, hopelessness, and guilt (Brown and Beail, 2009), 5) diminished psychological and social development, social isolation and exclusion, and institutionalization (Emerson et al. 1994), 6) neglect and abuse (Emerson et al. 1994), and 7) obstacles in receiving adequate care (Cowley et al. 2005). SIB also has great impact on families and professionals, such as feelings of anger, inadequacy and guilt (Fish 2000), and negative psychosocial effects (Mossman et al. 2002). Besides these personal consequences, SIB can lead to costly services and management difficulties (Emerson et al. 2001; Hassiotis et al. 2008), because of the required increased support or even one-to-one supervision (Tureck et al. 2013).

SIB is often persistent over time (Emerson et al. 2001; Kiernan and Alborz 1996; Murphy et al. 1993) and the occurrence of specific forms is relatively stable within the group showing severe SIB (Emerson et al. 2001). Persons with ID can show multiple forms of SIB (Emerson et al. 1997) and it can have a variety of etiologies (e.g., genetic, biological, psychological, environmental, or a combination) (Luiselli 2012). Associations have been found between SIB and several syndromes (e.g., Prader-Willi syndrome, Cri du Chat syndrome, Lesch Nyhan syndrome or Fragile X syndrome; Arron et al. 2011; Hall et al. 2001) and psychiatric conditions (e.g., borderline personality disorder, bipolar disorder, depressive disorder; Haw et al. 2001; Joyce et al. 2010; Zanarini et al. 2008).

Estimates of the prevalence of SIB in people with ID vary, partly due to the population and setting studied (Rojahn and Esbensen 2002) and the methodological diversity, but also due to the sometimes hidden nature of these behaviors (Nijman and à Campo 2002). Prevalence rates range between 4 and 23% in people with ID (Cooper et al. 2009; Kahng et al. 2002; Rojahn and Meier 2009).

There is increasing knowledge of causes and functions of SIB and the recognition for research to guide evidence-based interventions (Gratz 2003; Suyemoto 1998). Descriptive assessment and experimental functional analyses of SIB give a rich source of information about the setting and conditions (antecedents and consequences) that precede and follow SIB (Beavers et al. 2013). Beavers, Iwata, and Lerman (2013) showed in their review that SIB was mostly maintained by escape, attention and automatic reinforcement. Despite this relevant information, descriptive assessment and experimental functional analyses do not seem to be used often in clinical practice (Lydon, Healy, O’Reilly and Lang 2012). Lydon and colleagues note that performing functional analyses is not always easy in clinical practice, because it is time consuming, requires specific expertise to execute, and it is unsuitable for certain settings and types of behaviors. Another reason for this may be a lack of standardised and reliable ways to document incidents of SIB.

Indeed, when looking at the instruments used in research projects to assess the characteristics of SIB, it is found that most instruments are self-reports (e.g., the Self-Harm Inventory (SHI), Sansone et al. 1998) or informant based questionnaires (e.g., Self-Injurious Behavior Questionnaire (SIB-Q), Schroeder et al. 1997), and collect information about SIB in an indirect or retrospective way (Luiselli 2012; Sansone and Sansone 2010). These instruments do not primarily focus on the relation between the behavior of an individual and potential specific situational triggers in the environment of the self-injuring client, and thus may impede studying SIB in the context in which it occurs (place, time, direct trigger and reactions of the environment to the behavior). To our knowledge, only Nijman and à Campo (2002) and Tenneij and Koot (2008) conducted studies using the Staff Observation Aggression Scale-Revised (SOAS-R; Nijman et al. 1999a, 1999b), an incident based, observer assessed instrument for aggression. Using this aggression observation instrument, SIB (auto-aggressive behavior) can also be documented as a subsequent part of the observation.

To increase our knowledge about the situational determinants, triggers, and consequences of SIB, research is needed in which incidents of SIB are documented directly by observation in their natural context, using an instrument specifically designed to observe SIB. In this study we therefore focus on two goals. First to assess the clinical usefulness of an incident-based assessment instrument for SIB, called the ‘Self-Harm Scale’ (SHS). Second to provide more insight in the characteristics of SIB of people with ID and comorbid psychopathology as well as its frequency and severity, using an instrument focusing solely on SIB.

## Method

### Setting and Participants

This study was carried out in collaboration with three closed units of a treatment center, specialised in care for people with mild ID or borderline intellectual functioning and co-occurring psychiatric and/or behavior problems. Each unit contained 10 beds. Of a total of 40 support staff who completed the Self-Harm Scale (SHS), 63% (*n* = 25) were women. The average age of these 40 support staff members was 34.2 years (*SD* = 9.4). On average, they worked 7.1 years (*SD* = 6.8) with persons displaying self-injurious and/or other challenging behaviors. In the 41-week period (9 months) of data collection, 51 clients stayed at the centre. A total of 33 clients (64.7%) were included in the study. Clients were not included if a) they did not gave informed consent, b) they stayed for a period shorter than four weeks or c) the responsible psychologist did not agree the client took part in the study, because informing the client about the research and asking him/her for permission would possibly worsen the well-being of the client. Of the total participating group (*n* = 33), 20 persons (61%) were men. The mean age of the 33 participating clients was 31.3 years (*SD* = 11.4), the 33 participants had an average IQ score of 73 (*SD* = 6.4) and the average length of stay was 54 weeks (*SD* = 38.2).

Of the 33 clients, eleven persons (33%) were involuntary admitted, twelve persons (36%) were diagnosed with schizophrenia or other psychotic disorders, 7 persons (21%) with a mood disorder, 6 persons (18%) with a pervasive developmental disorder, 4 persons (12%) with an anxiety disorder and 4 persons (12%) had a different diagnosis (e.g., attention deficit disorder). In addition, 7 persons also had a personality disorder (21%).

## Materials

### The Self-Harm Scale

The Self-Harm Scale was initially developed by author 2 and 3. The two authors made a first draft of this scale based on their clinical experience with clients who display SIB in general psychiatric hospitals (e.g., see Nijman and à Campo 2002; Nijman et al. 1999a) and their expertise with developing incidents-based instruments regarding aggressive behavior. Following this, the first draft of the scale was presented to the members of the European Violence in Psychiatry Research Group (EViPRG), during a routine meeting of this group in Dublin several years ago for feedback. This resulted in several revisions and additions of some commonly used self-injuring methods that are seen in clinical practice on psychiatric wards, which were added to the second column of the SHS. The current study, however, is, as far as we know, the first study in which the SHS was used to document incidents of SIB in clinical practice. The current version of the SHS consists of five columns. The SHS provides the informant with selected options in separate boxes that only have to be marked by the respondents, such as place of self-harm: arm, neck or leg. In the first column of the SHS, support staff record which people were present during the incident, the location where the incident took place and what apparently triggered the SIB. The means used to self-injure are documented in the second column (e.g., using parts of the body (e.g., nails or head) and/or materials (e.g., knife or chemicals) to self-injure). In the third column support staff indicate the part(s) of the body that were involved in the SIB. In the fourth column, support staff list the consequences of the SIB for the person him- or herself. In the fifth and last column of the SHS, support staff document in what way the SIB stopped. This could be without intervention (e.g., client stopped the SIB by him- or herself) or by an intervention of support staff (e.g., verbal intervention or held with force). The SHS form is presented in the appendix of this article. Subsequently, support staff had to judge the overall severity of the SIB on a 100-mm Visual Analogue Scale (VAS), ranging from 0 (‘not severe at al’) to 100 (‘extremely severe’). Each support staff who witnessed SIB or was informed the client had injured him or herself was asked to complete a SHS.

### The Vineland-Z

The Dutch translation of the Vineland Adaptive Behavior Scales-Survey Form (VABS; de Bildt and Kraijer 2003), the Vineland-Z, was used to measure the adaptive behavior of the participants with ID. On the VABS, 225 items divided in three domains have to be completed, which concern: communication (*n* = 67), daily living skills (*n* = 92) and socialisation (*n* = 66). In an interview support staff indicate for each VABS-item whether their client usually performs in this way (score 2), sometimes or partly performs in this way (score 1), or never performs in this way (score 0). The total score of the list is the sum of all item scores, in which high scores represent higher levels of adaptive behavior. The instrument has a good reliability and validity in a population of people with ID (de Bildt et al. 2005).

## Procedure

Ethical approval to conduct the study was obtained both by the ethical committee of Tilburg University (EC-2013.30) and the participating treatment facility in the south-east of the Netherlands (2013.016.wk), in compliance with the Helsinki Declaration. Data collection took place between April 2014 and January 2015 (a 41-week period). Before data collection started, each person with ID (or their legal representative) that received treatment at the participating center during the observation period as well as their support staff were asked to join the study. Of all participants (i.e., persons with ID and support staff) who gave informed consent demographic variables were collected. Following this, the data collection with the SHS started. Support staff completed the SHS every time they witnessed or were informed about SIB of a client within the 41-week period. Finally, the two clients who showed most SIB and their psychologists were asked about their views on the SIB.

## Analysis

The characteristics of the people with ID and support staff, and the characteristics of the incidents were analysed using descriptive statistics in the Statistical Package for the Social Sciences (SPSS) version 22. A univariate comparative analyses with t-tests or chi-square calculation and, if needed, Fisher Exact tests (when the *n* was too small to perform a chi-square test), were conducted to investigate the potential differences between persons who had engaged in SIB and those who had not. Next, because two clients displayed the majority of the incidents, these two cases were analysed in detail, looking at all different variables (e.g., time of the incidents of SIB, triggers, consequences and location of SIB). Besides using statistical analyses, we screened the interviews of the psychologists and client on qualitative information related to the SIB. Quotes about the preceding events, the SIB, the consequences and measures that typified the SIB of the clients were subsequently extracted from the interviews, and added to the result section.

## Results

### Overall characteristics of SIB

Eight of the 33 persons with ID included in the study (24%) displayed some form of SIB based on the SHS forms. During the 41-weeks of data collection a total of 104 incident forms were completed by support staff of the three wards. The average number of SIB incidents was 2.5 per week, which would equal 4.0 incidents per participating client per year. In Table 1, the characteristics of the persons with ID who display SIB are summarised and compared with the persons with ID who did not show any form of SIB. Clients who did show SIB were a) more often female; b) more often diagnosed with a personality disorder (PD) and c) had better communication skills compared to the clients without SIB.

**Table 1:** Characteristics of persons with and without self-injurious behavior

	Self-injuring clients (*n* = 8)	Non-self-injuring clients (*n* = 25)	Statistical comparison	*P*
Gender, male: *n* (%)	1 (12.5)	19 (76.0)	Fisher Exact test	.003
IQ: mean (*SD*)	73.1 (7.7)	73.4 (6.1)	*t*(28) = −0.105	.917
Age, years: mean (*SD)*	30.9 (15.3)	31.5 (10.2)	*t*(9.083) = −0.107	.917
Diagnosis axis I, *n* (%)				
Schizophrenia or psychotic disorder	1 (12.5)	11 (44.0)	Fisher Exact test	.206
Pervasive developmental disorder	3 (37.5)	3 (12.0)	Fisher Exact test	.137
Mood disorder	2 (25.0)	5 (20.0)	Fisher Exact test	1.000
Anxiety disorder	2 (25.0)	2 (8.0)	Fisher Exact test	.241
Other disorder (e.g., Attention Deficit Disorder or substance-related disorder)	0 (0.0)	4 (16.0)	Fisher Exact test	.550
Diagnosis axis II, *n* (%)				
Personality disorder	4 (50.0)	3 (12.0)	Fisher Exact test	.042
Involuntary admitted, *n* (%)	3 (37.5)	8 (32.0)	Fisher Exact test	1.000
Length of admission: mean (*SD*)	52.7 (37.9)	54.4 (39.1)	*t*(31) = −0.110	.913
Adaptive behavior age: mean (*SD*)				
Communication	11.5 (1.1)	9.8 (2.1)	*t*(23.312) = 3.107	.005
Daily living skills	10.1 (1.9)	9.8 (2.5)	*t*(30) = 0.322	.750
Socialisation	8.0 (2.2)	6.6 (1.9)	*t*(31) = 1.714	.097
Total score on Vineland-Z	10.0 (2.0)	8.9 (2.1)	*t*(30) = 1.313	.199

The eight clients displaying SIB used various methods to injure themselves of which cutting was the most used method (*n* = 65, 63%), followed by head banging (*n* = 47, 45%), taking chemicals or medication (*n* = 12, 12%), injuring themselves by hitting against objects (*n* = 10, 10%) and strangulation (*n* = 8, 8%). In 33% (*n* = 34) of the incidents of SIB clients used more methods at the same time. In 31% (*n* = 32) of the incidents of SIB support staff indicated that they did not understand what triggered the SIB. In case support staff could specify what triggered SIB, stress inducing interactions (e.g., interactions between client and support staff, hearing bad news; *n =* 31, 30*%*) and a psychological state (e.g., dissociative state, traumatic flashbacks; *n =* 34, 33*%*) were the most frequently mentioned triggers. In 64 incidents of SIB (62%), no or only ‘minor’ consequences were registered, which are defined as a (physical) consequence that did not require medical assistance, like scratches. In 38% of the incidents (*n* = 40), however, the SIB resulted in more severe injuries, where medical assistance was required, like skin burns or unconsciousness. The average severity score (VAS) for the total group of participants who displayed SIB was 4.9 (SD 1.9, range 0.4 – 9.9). The severity scoring of support staff differed significantly between no or minor injuries and severe injuries (*t* (101) = −2.844, *p* = .005). That is, support staff experienced incidents of SIB as more severe (*M* = 56.1, *SD* = 16.2) if the consequences were also more severe according to the SHS, compared to incidents of SIB with no or minor consequences (*M* = 45.5, *SD* = 19.5). When intervening (84%), support staff used manual restraints most often (56%; *n* = 49), such as holding the arms of the clients to prevent the client from (further) cutting. In 36% (*n* = 31) support staff used verbal techniques or approached the client to stop the behavior, such as asking the client to stop immediately with SIB. In 17% (*n* = 18) of the incidents of SIB stopped without an intervention.

### Temperospatial characteristics of SIB

In line with an earlier study of Nijman and à Campo (2002), SIB most often took place in the bedroom of the client (93%, *n* = 97). The frequency of SIB differed significantly over the days [*χ* ^*2*^ (6) = 19.2, *p* = .004], with the highest number of incidents on Mondays (23%, *n* = 24). Most of the incidents occurred between 6.30 and 10.30 PM (57%, *n* = 59) [*χ* ^2^ (3) = 68.3, *p* < .001].

### SIB illustrated by two cases

Two of the eight clients who displayed SIB, which will be called Ms. M. and Ms. L., caused more than 80 % of the incidents (85%). The characteristics of these two clients are described in the next paragraph, completed with quotations from the interviews with M., the psychologist of M. and the psychologist of L.

### Description of SIB of M

M. is a 20-year-old woman (full-scale IQ = 67) diagnosed with a dissociative disorder, post-traumatic stress disorder and borderline personality disorder. She stayed at the treatment center for 77-weeks. In the 41-week of data collection, a total of 55 unique incidents of SIB were recorded, consisting mostly of head-banging (*n* = 35; 64%) and cutting (*n* = 33; 60%). Most of M.’s incidents of SIB took place in the evening, between 6.30 and 10.30 PM (80%; *χ*^2^ (3) = 89.1, *p* < .001), and were performed in her own room (96%). In 31% (*n* = 17) of the incidents support staff reported that the reason for the SIB was unclear to them. In the case support staff could indicate potential triggers that led to SIB, they often (*n* = 19, 50%) gave psychological reasons (e.g., emotions, dissociation, traumatic flashbacks) and three times (8%) the SIB was reported to follow an EMDR-treatment session. As the psychologist stated:

*We saw her glance changing, we could not make any contact any more, she (M.) did not response to her name….. Stress was the greatest trigger between not hurting and hurting herself.*

In 42% (*n = 16)* of the incidents, support staff indicated that a specific interaction appeared to have led to SIB. M.’s psychologist:

*Most of the time it was a longer course, during which you could see the tension rising. Conflicts with other clients, or trouble with support staff, when they had different opinions or when plans changed, disappointments, stuff with parents….eventually led to M. hurting herself.*

In 78% of the incidents SIB resulted in injuries, of which 40% were mild injuries (i.e., injuries for which no medical treatment was necessary such as scratches and bruises) and 60% major injuries (i.e., injuries for which medical assistance was necessary such as unconsciousness, vomiting and deep cuts). In 18% (*n* = 10) of the incidents support staff did not need to intervene to stop the SIB. In the other incidents intervening was required. In the majority of the incidents of SIB (80.0%), support staff manually restrained the client to stop the SIB. As the psychologist said:

*The only thing that you could do….literally…was to overpower her and prevent her from hurting herself further*.

In four of the incidents (9%) support staff also offered an alternate sensory stimulus (i.e., lemon juice) next to the interventions. That M.’s SIB at times was very severe, both for M. and the support staff, is illustrated by quotes from M.’s psychologist:

*It is a miracle that she is not…., that she did not decease, while she was doing this. She did a great appeal on the entire support staff, it continuous 24-hours a day. Sometimes I felt truly powerless.*

*I believe there was a lot of shame, and also fear for M. when she hurts herself*.

The view of M. on her behavior:

*Most of the time it happened in the evening, because I have a trauma about something that happened in the evening. I did it to get out of the traumatic flashbacks, it helped me to avoid thinking about the periods I have been through. Sometimes I just walked up and down, did not know what to do, and then I took a knife and cut myself. If I did not have anything, like in seclusion, I banged with my head. I often felt that a kind of ease came over me, which brought me to a normal level, so to say. Distractions helped me. Playing games, hitting a punching bag, sometimes just a hug and staying next to me, smoking cigarettes did help, but the best was just to offer closeness and seek distraction.*

### Description of SIB of L

L. is a 19-year-old woman (full-scale IQ = 79) diagnosed with a dysthymic disorder. She stayed at the treatment center for 29 weeks. In the 29-week of her stay, 33 incidents of SIB were recorded, of which 85% (*n* = 28) included cutting and 21% (*n* = 7) included head-banging. Most of the incidents occurred between 10.30 AM - 2.30 PM (61%; *χ*^2^ (3) = 24.3, *p* < .001) and were performed in her own room (91%). As her psychologist said:

*It most often happened in the beginning of the afternoon; …and during unoccupied moments [with no activities and distractions] which were also a problem for her*.

In 33% (*n* = 11) of the incidents support staff reported that the reason for the SIB was unclear to them. In the incidents support staff could identify potential triggers leading to SIB, they often (55%) indicated that a specific interaction led to SIB and in 41% they gave a psychological reason (e.g., being overwhelmed by emotions). In some cases, a trigger could be the sound of a train passing by. The psychologist of L.:

*If she saw a train, she thought about the train she once stood in front of when she wanted to commit suicide or she thought about… apparently there was a situation in which she was bullied…and some boys told her: ‘You are worthless, jump in front of a train’. It often was a kind of social situation or memory or flashback of a situation that triggered her, that got her out of balance and got her extreme tensioned, and it seemed she could not do anything else to regulate this tension than hurting herself.*

In all but one of the SIB incidents of L. (97%), the SIB resulted in injuries, of which 75% concerned minor injuries and 25% more severe injuries. When support staff felt they had to intervene, in 69% (*n* = 20) of the incidents the behavior stopped after verbal intervention or approaching of support staff. In 31% (*n* = 9) SIB was stopped by manually restraining L.

### Comparison of the two cases

**Fig. 1:**
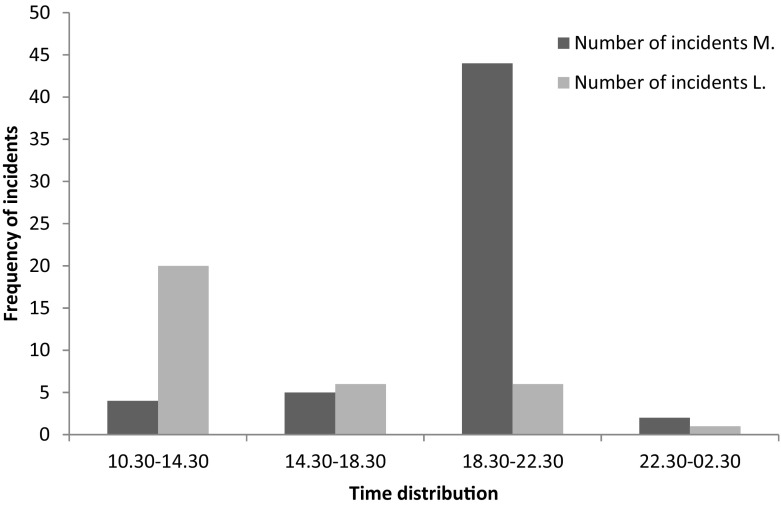
Frequency of incidents distributed over time

M. and L. differed in several aspects regarding their SIB. Most markedly where the differences in the time of the day on which the SIB took place (see Fig. 1) and the forms of SIB, consequences of and measures to stop SIB. There is a significant difference between M. and L. regarding the time of SIB. More specifically, L’s SIB occurred more often between 10.30 AM and 2.30 PM and that of M’s between 6.30 – 10.30 PM (*χ*^2^ (3) = 36.8, *p* < .001). The nature of SIB, mostly head-banging for M. and cutting for L., also impact the injuries and ways support staff tried to stop SIB. The injuries of M. were more often severe and support staff used more severe interventions to stop the SIB of M. compared to the SIB of L.

## Discussion

The goal of this study was twofold: 1) to assess the clinical usefulness of the Self-Harm Scale, and 2) to explore the characteristics, frequency and severity of SIB of people with ID and comorbid psychopathology. The results of this study indicate that SIB is a common problem in people with mild to borderline ID with 4.0 incidents per participating client annually, although a minority of clients were involved in engaging in SIB in the current study. This mean number of incidents per client per year is higher compared to earlier studies conducted with the same population (2.3 incidents per participating client per annum; Tenneij and Koot 2008) as well as with other populations (e.g., psychiatric patients; 0.3 per participating client annually; Nijman and à Campo 2002). In line with earlier studies (Nijman and à Campo 2002; Tenneij and Koot 2008), our research shows that the majority of the incidents were caused by a minority of people, and that the SIB incidents relatively often happened in the evenings. However, the two cases presented in the current study showed that the times on which clients engage in SIB can differ substantially. Furthermore, almost all incidents happened in the clients own room, which confirms earlier research stating that SIB often takes place in private places (Nijman and à Campo 2002). For support staff it was sometimes hard to determine what triggered the SIB, but most of the times interpersonal and intrapersonal motivations for the client were the trigger for SIB, as also becomes clear in other studies relating to SIB (Walsh 2006). As can be seen in the case-studies, it is important to consider both functions, as this can differ between clients but also within a client. Support staff most often used manual restraint to stop the SIB, which seems to be partly contrary to the findings of earlier research of for example Tenneij and Koot (2008) in which staff most often talked to the client to stop the SIB, and manual restraint was only used in 5% of the SIB-incidents. The SHS scale in our study is more purely focussed on physical self-injuring acts that have the aim to injure one’s own body, compared to the SOAS-R used by Tenneij and Koot, which is also used to document verbal (auto)-aggressive acts. The physical nature of the self-injuring acts that are documented with the SHS will make a direct physical response including manual restraints by staff more often necessary to prevent clients from further injuring themselves.

Comparison between clients with and without SIB revealed three significant differences. To the best of our knowledge, the association between female gender and SIB has not been reported in earlier studies with people with ID (e.g., McClintock et al. 2003), though it was found in a psychiatric sample showing SIB (Nijman and à Campo 2002). The association between SIB and BPD is also found in earlier research regarding psychiatric patients (Nijman and à Campo 2002; Zanarini et al. 2008). The association between communication skills and SIB is not in line with previous research, which reported that SIB is related to poor communication skills (Lowe et al. 2007). There are several reasons that might explain these differences between our findings and earlier research such as the small sample size and the collection of data in only one treatment facility in the current study as well as the differences in target population. The respondents in our study who displayed SIB were all people with ID and comorbid psychopathology. There was a significant difference between the self-injuring and non-self-injuring group related to having a personality disorder. The group who displayed SIB were more often diagnosed with a personality disorder. In our clinical experience clients with personality disorder socially interact and communicate with their environment much more than for example people with autism or psychotic disorders, who relatively often withdraw themselves from social interactions. As such, communication skills seem to be related to SIB, and as such may be an important variable to consider in the assessment and treatment of SIB. Further research on a larger scale, within multiple treatment centers, with the SHS may help to make the picture clearer.

In our opinion, the cases of M. and L. strongly suggest that it is important to analyse and translate the results of the structured clinical assessments of SIB incidents into an individualised approach and treatment. As can be seen in earlier research (Tenneij and Koot 2008), a minority of clients (8 out of 33 clients; 24%) were involved in all of the 104 reported incidents of SIB. Learning more about the specific characteristics of their repetitive SIB, and the circumstances under which the SIB occurs, may help to intervene more appropriately at the client level, which in turn can help to reduce the danger and devastating effects of these behaviors. In the cases of M. and L. the striking difference in when the SIB occurs (in the evening versus the morning), and the differences in what support staff noted on the SHS forms as the potential triggers of the SIB of M. and L., suggests that the causes and triggers for the SIB are different and urge different preventive strategies for these two clients. In line with earlier findings from Nijman and à Campo (2002), the risk of engaging in SIB in M. seems to be increased when she is at her room alone at night, without much distracting stimuli, which possibly gives room for memories and intrusions about earlier traumatic experiences. In the case of L. it appears that other conditions play a role in triggering her SIB. Especially when there is a lot of interaction and activities in the morning, she seems to become vulnerable for engaging in SIB. For intervening, this may imply that M. needs to be kept in contact and have some (distracting) activities or interactions during the evening to prevent SIB, whereas for L. overstimulation and unexpected situations during the daytime may need to be reduced. Moreover, the results of the current study illustrate in our opinion the importance of structured clinical assessments of SIB in getting to know more about this behavior, its client-specific triggers and consequences.

The present study can be seen as a first step towards gaining more insight in SIB of people with ID using an incident based SIB scale, such as the SHS. In line with clinical screening instruments for aggressive behavior of clients with ID (van den Bogaard et al. 2018), the SHS is an easy to use instrument for structured clinical assessment of SIB by support staff. Data of the SHS gives an overall picture of the SIB that takes place on a ward, with minimal time investment. The instrument identifies which clients perform SIB, and gives insight in the types and severity of SIB these clients display. Based on the data of the SHS, an in-depth analysis of the SIB of specific clients can be executed, using for example a functional behavior assessment, in which variables like setting events, duration (e.g., Gratz 2001) and onset of the behavior (e.g., Walsh 2007) can also be taken into account. In other words, the SHS can be seen as a screening instrument for SIB of clients with ID, and helps determining which clients cause most incidents and the main characteristics of their SIB. A functional analysis can be conducted to explore the functions, setting events and maintaining variables of SIB in specific clients. Subsequently, the SHS can then be used again as a useful outcome measure, to evaluate the effects of interventions that are derived from the functional analyses, and to indicate the effects of the chosen interventions on the amount of SIB displayed.

Further research is needed to study SIB, using the SHS, in larger samples from different populations and settings and to study the psychometric aspects of the SHS, to get a clearer picture on the reliability (e.g., the inter-observer agreement of the SHS) and to compare the results of the SHS with other measures of auto-aggressive behavior. Further research should also be conducted to give an indication of the clinical utility and the way this scale can be used together with functional assessment instruments. It is hoped that the SHS will help to gain more information about SIB and to design and test individualised intervention strategies for clients who repeatedly engage in SIB.
